# Development and validation of an interpretable neural network for prediction of postoperative in-hospital mortality

**DOI:** 10.1038/s41746-020-00377-1

**Published:** 2021-01-08

**Authors:** Christine K. Lee, Muntaha Samad, Ira Hofer, Maxime Cannesson, Pierre Baldi

**Affiliations:** 1 Department of Anesthesiology and Perioperative Care, University of California Irvine, Irvine, CA USA; 2 Department of Biomedical Engineering, University of California Irvine, Irvine, CA USA; 3 Department of Computer Sciences, University of California Irvine, Irvine, CA USA; 4 Department of Anesthesiology and Perioperative Medicine, University of California Los Angeles, Los Angeles, CA USA

**Keywords:** Risk factors, Outcomes research

## Abstract

While deep neural networks (DNNs) and other machine learning models often have higher accuracy than simpler models like logistic regression (LR), they are often considered to be “black box” models and this lack of interpretability and transparency is considered a challenge for clinical adoption. In healthcare, intelligible models not only help clinicians to understand the problem and create more targeted action plans, but also help to gain the clinicians’ trust. One method of overcoming the limited interpretability of more complex models is to use Generalized Additive Models (GAMs). Standard GAMs simply model the target response as a sum of univariate models. Inspired by GAMs, the same idea can be applied to neural networks through an architecture referred to as Generalized Additive Models with Neural Networks (GAM-NNs). In this manuscript, we present the development and validation of a model applying the concept of GAM-NNs to allow for interpretability by visualizing the learned feature patterns related to risk of in-hospital mortality for patients undergoing surgery under general anesthesia. The data consists of 59,985 patients with a feature set of 46 features extracted at the end of surgery to which we added previously not included features: total anesthesia case time (1 feature); the time in minutes spent with mean arterial pressure (MAP) below 40, 45, 50, 55, 60, and 65 mmHg during surgery (6 features); and Healthcare Cost and Utilization Project (HCUP) Code Descriptions of the Primary current procedure terminology (CPT) codes (33 features) for a total of 86 features. All data were randomly split into 80% for training (*n* = 47,988) and 20% for testing (*n* = 11,997) prior to model development. Model performance was compared to a standard LR model using the same features as the GAM-NN. The data consisted of 59,985 surgical records, and the occurrence of in-hospital mortality was 0.81% in the training set and 0.72% in the testing set. The GAM-NN model with HCUP features had the highest area under the curve (AUC) 0.921 (0.895–0.95). Overall, both GAM-NN models had higher AUCs than LR models, however, had lower average precisions. The LR model without HCUP features had the highest average precision 0.217 (0.136–0.31). To assess the interpretability of the GAM-NNs, we then visualized the learned contributions of the GAM-NNs and compared against the learned contributions of the LRs for the models with HCUP features. Overall, we were able to demonstrate that our proposed generalized additive neural network (GAM-NN) architecture is able to (1) leverage a neural network’s ability to learn nonlinear patterns in the data, which is more clinically intuitive, (2) be interpreted easily, making it more clinically useful, and (3) maintain model performance as compared to previously published DNNs.

## Introduction

We and others have recently shown that deep neural networks (DNNs) and random forest algorithms, using only readily available information extracted from the electronic health record before or at the end of surgery, can successfully predict postoperative in-hospital mortality with area under the curve (AUC) ranging from 0.87 to 0.93^1–3^. While DNNs and other machine learning models often have higher accuracy than simpler models like logistic regression (LR), they are often considered to be “black box” models and this lack of interpretability and transparency is considered a challenge for clinical adoption^4^. In healthcare, intelligible models not only help clinicians to understand the problem and create more targeted action plans, but also help to gain the clinicians’ trust. Thus, LR models remain popular in the healthcare space, as they are easily interpretable, robust, easy to implement, and usually have good performance, as previously observed in our work comparing DNNs to LR^3^. However, LR can be limited by the fact that it is a shallow model with no ability to create new feature representations, such as with DNNs. An LR model can only combine the input features linearly before passing that combination through a logistic function, and this linear combination of features may not reflect clinical intuition. For example, both hypervolemia and hypovolemia have been shown to increase the risk of postoperative complications, reflecting a nonlinear relationship between a patient’s volume status and the risk for complications^5^. Nonlinear relationships can be captured by LR, but only through extra featurization and analyses, which may result in an infinite number of possible relationships and combinations of features. While DNNs are capable of learning nonlinear relationships between features on their own, they lack the interpretability of LR.

One method of overcoming the limited interpretability of more complex models is to use Generalized Additive Models (GAMs). Standard GAMs simply model the target response as a sum of univariate models. Caruana et al. demonstrated that GAMs which also included pairwise interactions of features could be applied to real healthcare problems such as pneumonia risk with interpretability and high accuracy^6^. Through a graphical representation of each model feature’s learned contribution to the predicted risk, the interpretable GAMs help to visualize learned patterns and identify new patterns in the data or confirm what clinicians already know. Inspired by GAMs, the same idea can be applied to neural networks through an architecture referred to as Generalized Additive Models with Neural Networks (GAM-NNs)^7^. In GAM-NNs, a network is built on top of each input feature (or each group of input features) and the output of these networks are linearly combined to produce the final regression or classification output. To incorporate a modest number of pairwise interactions, additional networks processing the corresponding pairs can also be included. Pairing of features was not assessed in this study to avoid cluttering the final interpretation. Bras-Geraldes et al. showed GAM-NNs could be used to predict mortality in the ICU with an AUC of 0.83, using 19 features from vital signs, lab values, demographics, admission information, and comorbidities^8^.

In short, models like DNNs allow for learning the more complex relationship between the input and class label. However, they are not as easily interpretable as LR. In this manuscript, we present the development and validation of a model applying the concept of GAM-NNs to allow for interpretability by visualizing the learned feature patterns related to risk of in-hospital mortality for patients undergoing surgery under general anesthesia.

## Results

### Patient characteristics

The data consisted of 59,985 surgical records, and the percent of occurrence of in-hospital mortality was 0.81% (*n* = 389) in the training set and 0.72% (*n* = 87) in the testing set. Patient demographics and characteristics of the training and testing datasets are summarized in Table 1.

**Table 1:** Training and testing dataset patient characteristics reported as number patients (%) or mean ± standard deviation. HCUP code description and distribution is shown only for those representing >1% of the training dataset.

	Train	Test
No. of patients	47,988	11,997
No. of patients with in-hospital mortality (%)	389 (0.81%)	87 (0.73%)
Age (years)	56 ± 17	56 ± 18
Estimated blood loss (cc)	95 ± 540	94 ± 410
Presence of arterial line (%)	8585 (17.9%)	2135 (18.0%)
Presence of pulmonary artery line (%)	1641 (3.4%)	430 (3.6%)
Presence of central line (%)	2444 (5.1%)	635 (5.3%)
ASA score (%)		
1	3023 (6.3%)	762 (6.4%)
2	17930 (37.4%)	4477 (37.3%)
3	23960 (49.9%)	5986 (49.9%)
4	2911 (6.1%)	735 (6.1%)
5	144 (0.3%)	30 (0.3%)
6	4 (0.01%)	0 (0%)
HCUP code description (%)		
UPPER GASTROINTESTINAL ENDOSCOPY, BIOPSY	3864 (8.05%)	965 (8%)
COLONOSCOPY AND BIOPSY	1693 (3.53%)	388 (3.2%)
LAMINECTOMY, EXCISION INTERVERTEBRAL DISC	1029 (2.14%)	287 (2.4%)
OTHER THERAPEUTIC PROCEDURES, HEMIC AND LYMPHATIC SYSTEM	1013 (2.11%)	247 (2.1%)
OTHER OR THERAPEUTIC PROCEDURES ON RESPIRATORY SYSTEM	985 (2.05%)	254 (2.1%)
INCISION AND EXCISION OF CNS	942 (1.96%)	255 (2.1%)
OTHER OR PROCEDURES ON VESSELS OTHER THAN HEAD AND NECK	932 (1.94%)	207 (1.7%)
OTHER THERAPEUTIC ENDOCRINE PROCEDURES	904 (1.88%)	258 (2.2%)
HIP REPLACEMENT, TOTAL AND PARTIAL	792 (1.65%)	186 (1.6%)
ARTHROPLASTY KNEE	768 (1.6%)	193 (1.6%)
OTHER OR THERAPEUTIC NERVOUS SYSTEM PROCEDURES	750 (1.56%)	181 (1.5%)
THYROIDECTOMY, PARTIAL OR COMPLETE	737 (1.54%)	172 (1.4%)
SPINAL FUSION	735 (1.53%)	150 (1.3%)
OTHER OR THERAPEUTIC PROCEDURES ON BONE	722 (1.5%)	195 (1.6%)
CONVERSION OF CARDIAC RHYTHM	720 (1.5%)	184 (1.5%)
HEART VALVE PROCEDURES	715 (1.49%)	186 (1.6%)
CHOLECYSTECTOMY AND COMMON DUCT EXPLORATION	700 (1.46%)	216 (1.8%)
ENDOSCOPIC RETROGRADE CANNULATION OF PANCREAS (ERCP)	663 (1.38%)	155 (1.3%)
KIDNEY TRANSPLANT	659 (1.37%)	194 (1.6%)
OTHER OR THERAPEUTIC PROCEDURES ON NOSE, MOUTH AND PHARYNX	653 (1.36%)	173 (1.4%)
OTHER HERNIA REPAIR	652 (1.36%)	178 (1.5%)
HYSTERECTOMY, ABDOMINAL AND VAGINAL	641 (1.34%)	155 (1.3%)
APPENDECTOMY	634 (1.32%)	147 (1.2%)
OTHER THERAPEUTIC PROCEDURES ON MUSCLES AND TENDONS	629 (1.31%)	154 (1.3%)
COLORECTAL RESECTION	609 (1.27%)	127 (1.1%)
INSERTION, REVISION, REPLACEMENT, REMOVAL OF CARDIAC PACEMAKER OR CARDIOVERTER/DEFIBRILLATOR	601 (1.25%)	128 (1.1%)
ABORTION (TERMINATION OF PREGNANCY)	587 (1.22%)	162 (1.4%)
TREATMENT, FRACTURE OR DISLOCATION OF HIP AND FEMUR	570 (1.19%)	155 (1.3%)
OTHER OR GASTROINTESTINAL THERAPEUTIC PROCEDURES	569 (1.19%)	124 (1%)
OPEN PROSTATECTOMY	554 (1.15%)	140 (1.2%)
DIAGNOSTIC BRONCHOSCOPY AND BIOPSY OF BRONCHUS	550 (1.15%)	131 (1.1%)
NEPHRECTOMY, PARTIAL OR COMPLETE	526 (1.1%)	124 (1%)

### Development of the model

The final hyperparameters for the GAM-NN model with Healthcare Cost and Utilization Project (HCUP) features consist of one hidden layer with 50 neurons hyperbolic tangent (tanh) activations (Table 2). The model was trained with dropout probability of 0.5 and L2 weight decay of 0.0001. The final hyperparameters for the GAM-NN model without HCUP features were the same except for an L2 weight decay of 0.001.

**Table 2:** Final model parameters for each Generalized Additive Models with Neural Networks (GAM-NNs) model with and without HCUP category description features.

	No. of features	No. of hidden layers	No. of neurons	Hidden layer activation	Dropout probability	L2 lambda
With HCUP features	88	1	50	tanh	0.5	0.0001
Without HCUP features	55	1	50	tanh	0.5	0.001

### Model Performance

All performance metrics reported below refer to the testing set (*n* = 11,997).

#### Performance metrics

Area under the receiver operating characteristic curve (AUC ROC) and average precision (AP) are summarized in Table 3. The GAM-NN model with HCUP features had the highest AUC 0.921 (0.895–0.95). Overall, both GAM-NN models had higher AUCs than LR models, however had lower APs. The LR model without HCUP features had the highest AP 0.217 (0.136–0.31).

**Table 3:** Area under the receiver operating characteristic curve (AUC ROC) and average precision (AP) with 95% CIs for the Generalized Additive Models with Neural Networks (GAM-NNs) and logistic regression (LR) models, with and without HCUP category description features.

Feature set	Model	AUC	AP
With HCUP features	GANN	0.921 (0.895–0.95)	0.176 (0.109–0.26)
	LR	0.912 (0.879–0.94)	0.207 (0.127–0.3)
Without HCUP features	GANN	0.912 (0.883–0.94)	0.197 (0.124–0.29)
	LR	0.906 (0.873–0.94)	0.217 (0.136–0.31)

#### Interpretability: Visualizing feature contributions

To assess the interpretability of the GAM-NNs, we visualized the learned contributions of the GAM-NNs and compared against the learned contributions of the LRs for the models with HCUP features.

**Fig. 1:**
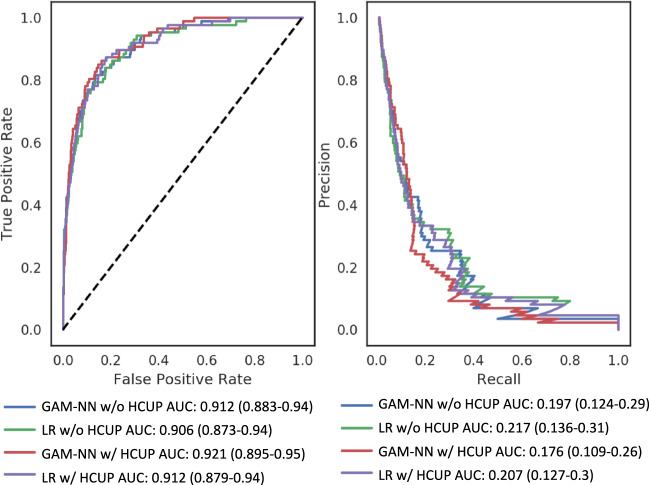
Receiver operator characteristic curves and precision-recall curves for LR models and GAM-NN for prediction of mortality with and without HCUP features. GAM-NN: Generalized Additive Models with Neural Networks; HCUP: Healthcare Cost and Utilization Project; LR: Logistic regression.

In Fig. 1, we visualize these contributions for a select sample of the top nine contributing features in the GAM-NN model. The top nine were chosen by selecting the features with the highest mean GAM-NN contribution. We did not include any binary features in this example, such as presence of arterial line, as their visualization would not be as interesting, since there would only be two values to plot.

We see that, overall, the direction of the learned contributions from both the GAM-NN and LR models were similar, i.e., as MAX_DES increases, the contributions for both models decreased. However, while the LR model will always have a linear relationship, the GAM-NN learned nonlinear relationships that were unique to each feature. For example, for the feature AVG_MAP_10_MIN we see a nonlinear function where GAM-NN contributions increase for mean arterial pressure (MAP) < 60 mmHg and MAP > 60 mmHg. One odd relationship is the one observed between ANES_CASE_HOURS and mortality risk, where, with less hours spent under anesthesia there was more contribution to mortality risk. This could be a reflection of the infrequency of extremely high anesthesia case hours (>10 h), and that in-hospital mortality patients may not spend significantly longer amounts of time under anesthesia compared to non-mortality patients. In addition, while risk contribution increased with lower MIN_DBP, there was the opposite relationship for AVG_DBP_10_MIN and AVG_DBP, which could indicate that not all summary measures of vital signs are the same, and that these should be taken into consideration when selecting features. Both of these examples demonstrate that the effect of a particular feature may not always represent an underlying physiological phenomena, and that modification of a particular feature for a particular patient may not necessarily produce a reduction in risk.

For an up-close comparison of interpretability at the patient-specific level, we can also look at the top GAM-NN contributors to the risk of mortality (Table 4). If we look at the top 10 GAM-NN contributions from the best-performing GAM-NN with HCUP features for two unique in-hospital mortality patients from the testing set, we can see that the features that contributed most were different. ASA was a top contributor for Patient Example 1 but not for Patient Example 2. Surgery-related features like presence of HCUP category 1 (HCUP_cat_1_YN) (Incision and excision of CNS), minimum case hemoglobin (MIN_HB), and time of anesthesia (ANES_CASE_HOURS) were top contributors for Patient Example 2, not found in Patient Example 1. While five of the shared top contributing features between Patient Examples 1 and 2 were blood pressure and phenylephrine-related features, Patient Example 1’s top contributing features also included an additional blood pressure and heart rate-related feature. These differences could indicate that while vital signs were top contributors for both patients, the surgery type contributed more to risk for Patient Example 2 than for 1.

**Table 4:** Top 10 neural network contributions learned from the best-performing Generalized Additive Models with Neural Networks (GAM-NNs) model with HCUP features, for two in-hospital mortality patient examples from the test set.

Patient Example 1 (top 10 contributions)			Patient Example 2 (top 10 contributions)		
Feature	Value	Contribution	Feature	Value	Contribution
ART_LINE_YN	1	0.993	HCUP_cat_1_YN (Incision and excision of CNS)	1	1.080
ASA_SCORE	4	0.939	ART_LINE_YN	1	0.993
MIN_DBP	22	0.269	MIN_DBP	19	0.271
AGE	81	0.259	MIN_HB	7.6	0.184
AVG_DBP	68	0.234	PHENYLEPHRINE_CURRENT_RATE_MCG_MIN	43	0.177
PHENYLEPHRINE_CURRENT_RATE_MCG_MIN	45	0.191	PHENYLEPHRINE_MAX_RATE_MCG_MIN	43	0.174
PHENYLEPHRINE_MAX_RATE_MCG_MIN	45	0.176	MIN_MAP	17	0.132
MIN_MAP	30	0.122	AGE	69	0.094
AVG_HR	95	0.104	AVG_DBP_10_min	72	0.043
AVG_DBP_10_min	74	0.060	ANES_CASE_HOURS	3.9	0.001

## Discussion

Despite their popularity and success in many applications such as speech recognition and computer vision, DNNs still face challenges to being fully accepted in the healthcare data space. There has been growing interest and success in the application of DNNs for healthcare tasks due to the availability of large and complex electronic biomedical data, such as genomic data, biomedical images, and electronic medical records (EMRs)^1–3,9–11^. In addition, in many cases, DNNs have shown better predictive performance than traditional models such as LR, however, a significant perceived problem with DNNs has been their “black box” reputation^4^. Clinicians are interested in not only the probability of an adverse event, such as in-hospital mortality, occurring but also need to understand what variables contributed to the increased risk so that they can change and target their therapies to potentially avoid an adverse event altogether. The inability of a model to allow for this level of transparency and interpretability is a potential barrier to positive clinical perception and can decrease trust and subsequently usability^12–14^. A small survey of ICU and ED clinicians found that clinicians viewed interpretability of a machine learning model as justification for clinical decision making following a model’s prediction, and so models should be built with enough transparency around the clinical features driving the model’s decision that clinicians could validate model outputs with their clinical knowledge and judgment^13^. Ginestra et al. found that when evaluating the real-time hospital implementation of their ML-based sepsis prediction alert, only 16% of providers found the alert helpful 6 h after an initial alert and only 9% reported that the alert changed management^14^. In addition, the most frequent suggestion by clinicians was transparency regarding factors leading to a sepsis alert. LR is often preferred in the medical field due to its easy implementation and interpretability. The learned coefficients can easily be extracted and interpreted as relative significance, and odds ratios calculated from those coefficients are routinely used in the medical research community to interpret a feature’s contribution to increased odds of an adverse event. However, LR is a shallow model with no ability to create new feature representations and can only combine the features linearly before passing through a logistic function to represent the probability of response labels, such as in-hospital mortality. Neural networks have the ability to self-learn new and significant linear and nonlinear features that are combinations of the original input features. However, these features can be thought of as “hidden” in the network layers. In this study, we were able to demonstrate that our proposed generalized additive neural network (GAM-NN) architecture is able to (1) leverage a neural network’s ability to learn nonlinear patterns in the data, which is more clinically intuitive, (2) be interpreted easily, making it more clinically useful, and (3) maintain model performance as compared to our first study^3^.

**Fig. 2:**
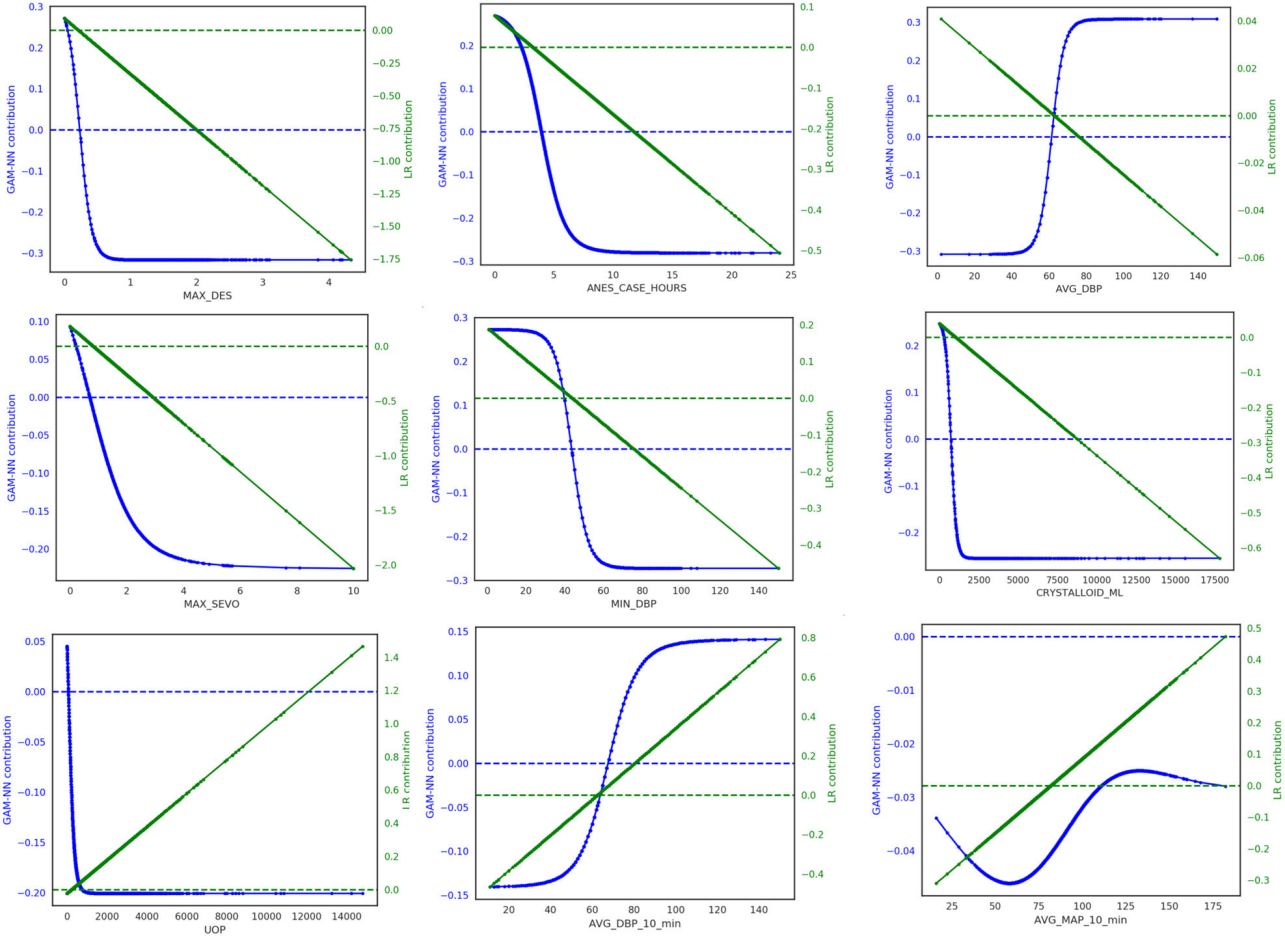
Sample of nine continuous features that had the highest mean mortality risk GAM-NN contributions across all patients, in order of highest to lowest. These features in order are maximum desflurane (MAX_DES), total anesthesia case hours (ANES_CASE_MINUTES), average diastolic blood pressure (AVG_DBP), maximum sevoflurane (MAX_SEVO), minimum diastolic blood pressure (MIN_DBP), total crystalloid administered (CRYSTALLOID_ML), urine output (UOP), average diastolic blood pressure of the last 10 min of the case (AVG_DBP_10_min), and average mean blood pressure of the last 10 min of the case (AVG_MAP_10_min). The feature’s values for all patients are plotted on the *x*-axis and the respective GAM-NN contribution (blue) on the primary *y*-axis and LR contribution (green) on the secondary *y*-axis. The more negative the risk contribution, the less contribution the respective value has to the risk of mortality.

It should be noted that LR models can still incorporate nonlinear feature representations, but this requires extra featurization. For example, hypotension and hypertension are both of concern during surgery. If we use the average MAP as a feature, an LR model would only learn a coefficient that indicates either risk increases with increased MAP or risk decreases with increased MAP, as we see in Fig. 2. To incorporate the domain knowledge that risk should increase with both high MAP and with low MAP, the MAP feature would have to be transformed into new features, i.e., binning the MAP values and creating multiple binary features. However, neural networks minimize the need for this type of tedious feature engineering and preprocessing, and they can effectively learn this clinically intuitive relationship without the domain knowledge or extra featurization.

Two limitations to our current study are that we were only able to develop and validate our model on (1) a single institution and (2) from the years 2013 to 2016, potentially limiting the generalizability of our results. Clinical practice not only varies from institution to institution, but also can change year to year with the emergence of new clinical evidence. While the difficulty in having large enough retrospective medical datasets to effectively train very complex models such as DNNs is no longer a limitation, developing the infrastructure to be able to not only gain access to the data, but to also obtain the data and process it for research use is a tremendous task. Obtaining past and new data from the same institution can itself be limiting, and the ability to access and integrate other institutions’ data for validation can also be difficult. One benefit of this model is that the features needed from other institutions to validate our model are not only common across all institutions, but are also commonly used. For example, MAP is a commonly observed vital sign, however, features like central venous pressure (CVP) and pulmonary arterial pressure (PAP) require invasive catheters and are only standard of care in more critical patients. Features like the bispectral index (BIS) do not require invasive catheters, however, it is not standard of care practice to monitor it. Thus, we expect the features in our model to be applicable to all patients across all institutions. However, it should also be noted that standard-of-care practice also varies from institution to institution, and so patterns discovered in this single-institution dataset may not be generalizable to other institutions and may require re-training of the model to individual institutions or more variety of institutions. As mentioned before, developing the infrastructure for such data extraction is a difficult process. The Perioperative Data Warehouse at UCLA^15^ used in the data extraction for this study exemplifies how this can be done successfully, however, replicating the process at another institution with a different electronic health record system and standardizing the disparate medical dataset to be able to merge it with our current one is a well-known issue in the medical data community. Despite the difficulties mentioned above in obtaining new data for validation, we are currently working to address the limitations of our current validation results by collaborating with other institutions to replicate the data extraction used in this study as well as working within our own institution to access more recent data to validate the generalizability of our model.

In addition, while the models in this study were made to be interpretable, it should be emphasized that the interpretation is not necessarily causation, and the modification of a highly contributing specific feature would not necessarily decrease the patient’s risk of mortality. For example, in Table 4, both patient examples have high contribution related to arterial line placement, but deciding to not place an arterial line would not necessarily result in avoiding mortality. This is also true of other models such as LR. Although our model is transparent and the extraction of feature contributions described here explains how the model made the predictions, the relationship between the features and the risk of in-hospital mortality should still be thought of as correlation. These relationships would likely change with the removal of various features or addition of new features. However, the relationships learned in this model appear to be clinically intuitive and they are still important in that they provide new or confirm known insight that is not usually available with DNNs.

While we are no longer limited to using more traditional methods such as LR due to availability of data when developing more complex models, we should consider the needs for clinical adoption and impact. DNNs, such as ours, can be automated and incorporated with real-time EMR data. For example, with our model, all the model input features described can easily be automatically extracted or calculated at the end of surgery and our model would then be used to provide a probability for in-hospital mortality. If the probability is high, a summary of which features contributed the most to an increased risk of mortality (Table 4) and where the patient lies relative to other patients (Fig. 2) can also be displayed for the clinician. Thus, our model can serve as clinical decision support tool helping to identify patients in need of more postoperative resources and potentially informing therapeutic actions. For example, if a patient’s minimum DBP being very low contributed the most to that patient’s high risk of in-hospital mortality, the clinician may consider hypotension and associated risks such as acute kidney injury. A very different application of our model would be to re-train and apply it to a single institution to understand what areas of a patient’s care during surgery clinicians could be paying more attention to moving forward, if they are not already. For example, in Fig. 2, low average MAP below 50 and high average MAP above 80 are both associated with increased risk of in-hospital mortality. Clinicians at this institution could then target therapies during surgery to never leave that range of MAP. However, at a different institution, the learned relationship could be different and the targeted range of MAP may change based on the current practices of that institution. In either application, our model could be used to quickly assess a large amount of data and provide actionable insight, a task that may otherwise be time-consuming for clinicians.

In summary, this study shows that DNNs can be made to be not only accurate, but also interpretable. Any complex predictive model needs both to build enough trust that a clinician can interpret and act on a model’s decision over or complementary to their own clinical intuition. Future work includes not only validating the performance and generalizability of this model on other hospitals’ datasets, but also assessing how clinicians interact with the interpretability of the model.

## Methods

This manuscript follows the “Guidelines for Developing and Reporting Machine Learning Predictive Models in Biomedical Research: A Multidisciplinary View”^16^.

### Electronic medical record data extraction and description

All data used in this study came from the UCLA Medical Center’s Perioperative Data Warehouse, a custom data warehouse built on top of the EMR (EPIC Systems, USA) and has been described in a previous paper^15^. All data used for this study were obtained from this data warehouse and IRB approval (UCLA-A IRB#15-000518) has been obtained for this retrospective review. Patients’ written approval was waived because of the retrospective nature of this study. Data included all surgical procedures performed between March 1, 2013 and July 16, 2016, and excluded cases not performed under general anesthesia, ambulatory cases, and patients older than 89 or less than 18 years of age.

### Model endpoint definition

The definition for in-hospital mortality was defined in the same way as described in our previous work^3^. The occurrence of an in-hospital mortality was extracted as a binary event [0, 1] based upon either the presence of a “mortality date” in the EMR between surgery time and discharge, or a discharge disposition of expired combined with a note associated with the death (i.e., death summary, death note). The definition of in-hospital mortality was independent of length of stay in the hospital.

### Model input features

The data and features used in this study are from our previous work modeling in-hospital mortality^3^. The data consists of 59,985 patients with an original feature set of 87 features extracted at the end of surgery. These features included demographics, labs, ASA score, intraoperative vital signs, total case time, medication administration, and anesthesia events. These original 87 features were reduced to 45 features in our previous work, and ASA was added as a feature in the final model (46 features) that improved model performance^3^. In this study, we used the same 46 features, and also added previously not included features: total anesthesia case time (1 feature); the time in minutes spent with MAP below 40, 45, 50, 55, 60, and 65 mmHg (6 features); and HCUP Code Descriptions of the Primary current procedure terminology (CPT) codes (33 features) (Table 5). There were 183 unique HCUP Code Descriptions in our dataset, and we selected 33 HCUP Code Descriptions that were present in at least 1% of the total data (Supplementary Table 1). These HCUP Code Descriptions were then encoded as 33 binary features.

**Table 5:** Description of model input features.

Feature	Feature description
AGE	Age of the patient in years (note we exclude ages <18 and >89)
ANES_CASE_HOURS	Case time under anesthesia in hours
ART_LINE_YN	Presence of arterial line
ASA_SCORE	ASA score
AVG_SBP, AVG_DBP, AVG_MAP, AVG_HR, AVG_PULSE_OX	Average systolic BP, diastolic BP, mean BP, heart rate, and pulse oximetry for the case
AVG_SBP_10_MIN, AVG_DBP_10_MIN, AVG_MAP_10_MIN, AVG_HR_10_MIN, AVG_PULSE_OX_10_MIN	Average systolic BP, diastolic BP, mean BP, heart rate, and pulse oximetry for the last 10 min of the case
BASELINE_GFR	Most recent GFR prior to surgery (only within 365 days)
COLLOID_ML	Total colloid administered
CRYSTALLOID_ML	Total crystalloid administered
CURRENT_HB, STARTING_HB	Most recent hemoglobin prior to surgery, Starting hemoglobin
CVC_ANES_YN	Presence of a central venous line
EBL	Total estimated blood loss
EPINEPHRINE_CURRENT_RATE_MCG_KG_MIN, EPINEPHRINE_MAX_RATE_MCG_KG_MIN	End of case rate of epinephrine, Highest infusion rate of epinephrine during the case
ESMOLOL_CURRENT_RATE_MCG_KG_MIN, ESMOLOL_MAX_RATE_MCG_KG_MIN	End of case rate of esmolol, Highest infusion rate of esmolol during the case
HCUP_CAT_x_YN	33 binary features for HCUP Category Descriptions IDs: [1. 3. 9. 10. 12. 33. 37. 42. 43. 48. 61. 67. 70. 76. 78. 80. 82. 84. 86. 99. 104. 105. 114. 124. 126. 146. 152. 153. 158. 160. 161. 172. 225]
MAX_DBP, MAX_DES, MAX_GLUCOSE, MAX_HR, MAX_ISO, MAX_MAP, MAX_PULSE_OX, MAX_SBP, MAX_SEVO	Maximum diastolic BP for the case, Maximum MAC of desflurane during the case (note this is not age adjusted), Maximum glucose for the case, Maximum heart rate for the case, Maximum MAC of isoflurane during the case (note this is not age adjusted), Maximum mean BP for the case, Maximum pulse oximetry for the case, Maximum systolic BP for the case, Maximum MAC of sevoflurane during the case (note this is not age adjusted)
MILRINONE_CURRENT_RATE_MCG_KG_MIN, MILRINONE_MAX_RATE_MCG_KG_MIN	End of case infusion rate of milrinone, Highest infusion rate of milrone during the case
MIN_DBP, MIN_GLUCOSE, MIN_HB, MIN_HR, MIN_MAP, MIN_PULSE_OX, MIN_SBP	Minimum diastolic BP for the case, Minimum glucose for the case, Minimum hemoglobin during the case, Minimum heart rate for the case, Minimum mean BP for the case, Minimum pulse oximetry for the case, Minimum systolic BP for the case
MIN_MAP_LT_40,MIN_MAP_LT_45,MIN_MAP_LT_50,MIN_MAP_LT_55,MIN_MAP_LT_60 min_MAP_LT_65	Minutes MAP < 40 mmHg, Minutes MAP < 45 mmHg, Minutes MAP < 50 mmHg, Minutes MAP < 55 mmHg, Minutes MAP < 60 mmHg, Minutes MAP < 65 mmHg
NICARDIPINE_CURRENT_RATE_MG_HR, NICARDIPINE_MAX_RATE_MG_HR	End of case rate of nicardipine, Highest infusion rate of nicardipine during the case
NITRIC_OXIDE_YN	Nitric oxide used for the case
NITROGLYCERIN_CURRENT_RATE_MCG_MIN, NITROGLYCERIN_MAX_RATE_MCG_MIN	End of case rate of nitroglycerin, Highest infusion rate of nitroglycerin during the case
NITROPRUSSIDE_CURRENT_RATE_MCG_KG_MIN, NITROPRUSSIDE_MAX_RATE_MCG_KG_MIN	End of case rate of nitroprusside, Highest infusion rate of nitroprusside during the case
PA_LINE_YN	Presence of pulmonary artery catheter
PHENYLEPHRINE_CURRENT_RATE_MCG_MIN, PHENYLEPHRINE_MAX_RATE_MCG_MIN	End of case rate of phenylephrine, Highest infusion rate of phenylephrine during the case
UOP	Total urine output
VASO_CURRENT_RATE_UNITS_HR, VASOPRESSIN_MAX_RATE_UNITS_HR	End of case rate of vasopressin, Highest infusion rate of vasopressin during the case
XFUSION_RBC_ML	Total red blood cells transfused

### Data preprocessing

Before model development, missing values for ASA scores were filled with the most common value (ASA 3); missing values for medications administration features indicated that no medication was actually administered and so were filled with 0; and all other missing values were filled with the means for that feature. Values that were greater than a clinically normal maximum (determined by M.C. and I.H.) were set to a maximum possible, as described in previous work^3^. Finally, all training data were rescaled to have mean 0 and standard deviation 1 per feature. Testing data were rescaled with the training data mean and standard deviation.

### Development of the model and feature contribution extraction

**Fig. 3:**
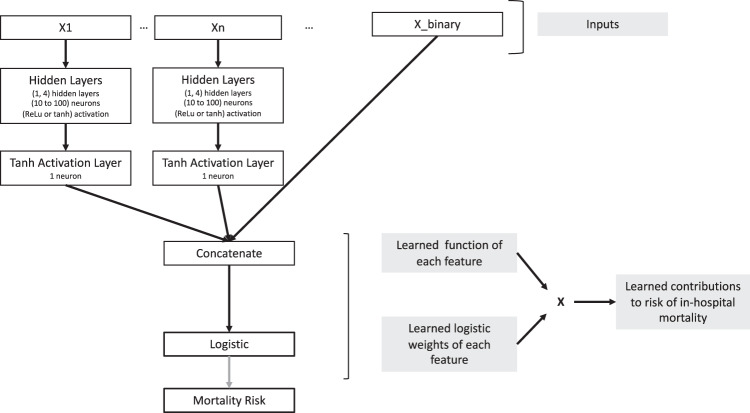
Proposed generalized additive models with neural networks architecture. This figure describes feature contributions calculation, for *n* individual continuous features (X1,…,X*n*) vs binary features (X_binary).

In this work, we were interested in classifying patients at risk of in-hospital mortality utilizing a proposed generalized additive neural network (GAM-NN) architecture (Fig. 3). All data were randomly split into 80% for training (*n* = 47,988) and 20% for testing (*n* = 11,997) prior to model development.

The loss function used in training was cross-entropy and to deal with the highly unbalanced classes, we applied class weights to the loss function by assigning the positive class 100x weight compared to the negative class to reflect the <1% occurrence of in-hospital mortality in our dataset.

To optimize hyperparameters, a grid search across varying hyperparameter combinations was performed, where each model was trained on 80% of the data with 5-fold cross validation. The model with the highest mean 5-fold validation AUC was chosen as the one with the best hyperparameter combination, and retrained on all of the training data prior to being tested. All hyperparameter values that were assessed are shown in parentheses. All models were trained with a batch size of 256 and Adam optimization^17^ with default parameters and reduced the learning rate by a factor of 10 when the validation loss stopped improving for five consecutive epochs, a batch size of 256 and a maximum of 100 epochs. Dropout (0.25, 0.5, 0.9)^18,19^ and L2 regularization (0.001, 0.0001) were also used to prevent overfitting. In our GAM-NN architecture, each feature had its set of hidden layers (1–4) with layer sizes of 10, 40–50, 90, 100 neurons with all activations being either rectified linear unit (ReLu) or hyperbolic tangent (tanh) (Fig. 3). These hidden layers are followed by a last layer with just one neuron with a tanh activation. This last tanh layer transforms the previous layer’s output into one value and forces the feature’s neural network final output to be between −1 and 1. The outputs of all the features’ tanh layers are then concatenated prior to being input into the final logistic layer (Fig. 3). The feature contributions are calculated as their tanh layer outputs multiplied by their respective logistic weights. Binary features only had a direct connection from the input layer to the final logistic layer, and so their feature contributions are calculated as the input value multiplied by their respective logistic weights.

### HCUP feature experiments

HCUP codes provide informative groupings in regard to a patient’s surgery and are also uniformly coded, making them easy to use as model inputs. However, they are not immediately available at the end of surgery, and so their inclusion could limit our model’s practical use. Thus, we also assessed developing a model without HCUP features to assess the impact on performance.

### Model performance

All model performances were assessed on the 20% of the data held out from training as a testing set. The same training and testing sets were used in this work as our previous work on in-hospital mortality for comparison^3^. Model performance was compared to a standard LR model using the same features as the GAM-NN.

### Performance metrics

Model performance was assessed using area under the receiver operating characteristic curve (AUC ROC) and average precision (AP), and 95% confidence intervals were calculated using bootstrapping with 1000 samples.

### Interpretability: Visualizing feature contributions

As previously described, the learned contribution of the GAM-NNs for each feature is its last tanh layer’s output multiplied by its respective weight from the logistic layer. Since the binary features have a direct connection from input to the logistic layer, the binary features’ learned contributions would be their input values multiplied by their respective weight from the logistic layer. For every data sample, each individual feature’s value was plotted on the *x*-axis vs its respective contribution on the *y*-axis. Individual feature contributions in the LR model were calculated as the individual feature’s value multiplied by its learned coefficient. For both models, the more negative the risk contribution, the less contribution the respective value has to the risk of mortality.

All neural network models were developed using Keras^20^. LR models and performance metrics were calculated with scikit-learn^21^.

### Reporting summary

Further information on research design is available in the Nature Research Reporting Summary linked to this article.

## Supplementary information

Supplementary Table 1

Reporting Summary

## Data Availability

The datasets generated during and/or analyzed during the current study are not publicly available due to institutional restrictions on data sharing and privacy concerns. However, the data are available from the corresponding author on reasonable request.

## Code availability

Code are available from the corresponding author on reasonable request. All neural network models were developed using Keras. LR models and performance metrics were calculated with scikit-learn.
