# Supplementary material for: Development and validation of an interpretable neural network for prediction of postoperative in-hospital mortality
Source: NPJ Digit Med. 2021 Jan 8;4:8. doi: 10.1038/s41746-020-00377-1 (PMC7794438; doi:10.1038/s41746-020-00377-1)
Supplement: Supplementary file 1 — Supplementary Table 1 [file 41746_2020_377_MOESM1_ESM.pdf]

Supplementary Table 1. Respective HCUP descriptions for each of the 33 HCUP category IDs included in the models

HCUP Category ID	HCUP Description
1	Incision and excision of CNS
3	Laminectomy; excision intervertebral disc
9	Other OR therapeutic nervous system procedures
10	Thyroidectomy; partial or complete
12	Other therapeutic endocrine procedures
33	Other OR therapeutic procedures on nose; mouth and pharynx
37	Diagnostic bronchoscopy and biopsy of bronchus
42	Other OR therapeutic procedures on respiratory system
43	Heart valve procedures
48	Insertion; revision; replacement; removal of cardiac pacemaker or cardioverter/defibrillator
61	Other OR procedures on vessels other than head and neck
67	Other therapeutic procedures; hemic and lymphatic system
70	Upper gastrointestinal endoscopy; biopsy
76	Colonoscopy and biopsy
78	Colorectal resection
80	Appendectomy
82	Endoscopic retrograde cannulation of pancreas (ERCP)
84	Cholecystectomy and common duct exploration
86	Other hernia repair
99	Other OR gastrointestinal therapeutic procedures
104	Nephrectomy; partial or complete
105	Kidney transplant
114	Open prostatectomy
124	Hysterectomy; abdominal and vaginal
126	Abortion (termination of pregnancy)
146	Treatment; fracture or dislocation of hip and femur
152	Arthroplasty knee
153	Hip replacement; total and partial
158	Spinal fusion
160	Other therapeutic procedures on muscles and tendons
161	Other OR therapeutic procedures on bone
172	Skin graft
225	Conversion of cardiac rhythm
