# Supplementary material for: Development and validation of an interpretable neural network for prediction of postoperative in-hospital mortality
Source: NPJ Digit Med. 2021 Jan 8;4:8. doi: 10.1038/s41746-020-00377-1 (PMC7794438; doi:10.1038/s41746-020-00377-1)
Supplement: Supplementary file 2 — Reporting Summary [file 41746_2020_377_MOESM2_ESM.pdf]

nature research

Corresponding author(s): Maxime Cannesson

Last updated by author(s): Oct 12, 2020

Reporting Summary

Nature Research wishes to improve the reproducibility of the work that we publish. This form provides structure for consistency and transparency in reporting. For further information on Nature Research policies, see our Editorial Policies and the Editorial Policy Checklist.

Statistics

For all statistical analyses, confirm that the following items are present in the figure legend, table legend, main text, or Methods section.

n/a	Confirmed	
☒	☐	The exact sample size (*n*) for each experimental group/condition, given as a discrete number and unit of measurement
☒	☐	A statement on whether measurements were taken from distinct samples or whether the same sample was measured repeatedly
☐	☒	The statistical test(s) used AND whether they are one- or two-sided<br>*Only common tests should be described solely by name; describe more complex techniques in the Methods section.*
☐	☒	A description of all covariates tested
☐	☒	A description of any assumptions or corrections, such as tests of normality and adjustment for multiple comparisons
☐	☒	A full description of the statistical parameters including central tendency (e.g. means) or other basic estimates (e.g. regression coefficient) AND variation (e.g. standard deviation) or associated estimates of uncertainty (e.g. confidence intervals)
☐	☒	For null hypothesis testing, the test statistic (e.g. *F*, *t*, *r*) with confidence intervals, effect sizes, degrees of freedom and *P* value noted<br>*Give P values as exact values whenever suitable.*
☐	☒	For Bayesian analysis, information on the choice of priors and Markov chain Monte Carlo settings
☐	☒	For hierarchical and complex designs, identification of the appropriate level for tests and full reporting of outcomes
☐	☒	Estimates of effect sizes (e.g. Cohen's *d*, Pearson's *r*), indicating how they were calculated

Our web collection on statistics for biologists contains articles on many of the points above.

Software and code

Policy information about availability of computer code

Data collection	R
Data analysis	R

For manuscripts utilizing custom algorithms or software that are central to the research but not yet described in published literature, software must be made available to editors and reviewers. We strongly encourage code deposition in a community repository (e.g. GitHub). See the Nature Research guidelines for submitting code & software for further information.

Data

Policy information about availability of data

All manuscripts must include a data availability statement. This statement should provide the following information, where applicable:
- Accession codes, unique identifiers, or web links for publicly available datasets
- A list of figures that have associated raw data
- A description of any restrictions on data availability

The datasets generated during and/or analyzed during the current study are not publicly available due to institutional restrictions on data sharing and privacy concerns. However, the data are available from the corresponding author on reasonable request.

Field-specific reporting

Please select the one below that is the best fit for your research. If you are not sure, read the appropriate sections before making your selection.

☒ Life sciences ☐ Behavioural & social sciences ☐ Ecological, evolutionary & environmental sciences

For a reference copy of the document with all sections, see nature.com/documents/nr-reporting-summary-flat.pdf

Life sciences study design

All studies must disclose on these points even when the disclosure is negative.

Sample size	The data consists of 59,985 patients with an original feature set of 87 features extracted at the end of surgery. These features included demographics, labs, ASA score, intraoperative vital signs, total case time, medication administration, and anesthesia events. All data was randomly split into 80% for training (n= 47,988) and 20% for testing (n=11,997) prior to model development.<br>The loss function used in training was cross entropy and to deal with the highly unbalanced classes, we applied class weights to the loss function by assigning the positive class 100x weight compared to the negative class to reflect the <1% occurrence of in-hospital mortality in our dataset.<br>To optimize hyperparameters, a grid search across varying hyperparameter combinations was performed, where each model was trained on 80% of the data with 5-fold cross validation. The model with the highest mean 5-fold validation AUC was chosen as the one with the best hyperparameter combination, and retrained on all of the training data prior to being tested. All hyperparameter values that were assessed are shown in parentheses.
Data exclusions	Data included all surgical procedures performed between March 1, 2013 and July 16, 2016, and excluded cases not performed under general anesthesia, ambulatory cases, and patients older than 89 or less than 18 years of age.
Replication	NA
Randomization	NA
Blinding	NA

Reporting for specific materials, systems and methods

We require information from authors about some types of materials, experimental systems and methods used in many studies. Here, indicate whether each material, system or method listed is relevant to your study. If you are not sure if a list item applies to your research, read the appropriate section before selecting a response.

Materials & experimental systems

n/a	Involved in the study	
☒	☐	Antibodies
☒	☐	Eukaryotic cell lines
☒	☐	Palaeontology and archaeology
☒	☐	Animals and other organisms
☐	☒	Human research participants
☒	☐	Clinical data
☒	☐	Dual use research of concern

Methods

n/a	Involved in the study	
☒	☐	ChIP-seq
☒	☐	Flow cytometry
☒	☐	MRI-based neuroimaging

Human research participants

Policy information about studies involving human research participants

Population characteristics	All data used in this study came from the UCLA Medical Center's Perioperative Data Warehouse, a custom data warehouse built on top of the electronic medical record (EPIC Systems, USA) and has been described in a previous paper.10 All data used for this study were obtained from this data warehouse and IRB approval (UCLA-A IRB#15-000518) has been obtained for this retrospective review. Data included all surgical procedures performed between March 1, 2013 and July 16, 2016, and excluded cases not performed under general anesthesia, ambulatory cases, and patients older than 89 or less than 18 years of age.
Recruitment	Data included all surgical procedures performed between March 1, 2013 and July 16, 2016, and excluded cases not performed under general anesthesia, ambulatory cases, and patients older than 89 or less than 18 years of age.
Ethics oversight	IRB approval (UCLA-A IRB#15-000518) has been obtained for this retrospective review.

Note that full information on the approval of the study protocol must also be provided in the manuscript.
